# Phenotype of sickle cell disease. Correlation of haplotypes and polymorphisms in cluster β, BCL11A, and HBS1L−MYB. Pilot study

**DOI:** 10.3389/fmed.2025.1347026

**Published:** 2025-02-12

**Authors:** Paloma Ropero, Miriam Peral, Luis Javier Sánchez-Martínez, Sara Rochas, Miguel Gómez-Álvarez, Jorge M. Nieto, Fernando A. González, Ana Villegas, Celina Benavente

**Affiliations:** ^1^Servicio de Hematología y Hemoterapia, Hospital Clínico San Carlos, Madrid, Spain; ^2^Instituto de Investigación Sanitaria Hospital Clínico San Carlos, Madrid, Spain; ^3^Instituto de Biología y Genética Molecular, Valladolid, Spain; ^4^Departamento de Biodiversidad, Ecología y Evolución, Facultad de Biología, Universidad Complutense de Madrid, Madrid, Spain

**Keywords:** sickle cell disease, cluster haplotypes β, *Xmn*I, BCL11A, HBS1L-MYB, fetal hemoglobin

## Abstract

**Objective/Background:**

Sickle cell disease (SCD) is a monogenic disease with a highly variable phenotype depending on the amount of fetal hemoglobin (HbF), the main modulator. Variation of HbF levels among patients is genetically regulated. HbF determines both the phenotype of the disease and the response to treatment with the main drug used, hydroxyurea. The efforts of the researchers have focused on discovering the genetic factors responsible for HbF variation, mainly describing the haplotypes of the β cluster and single nucleotide polymorphisms (SNPs) at three different loci: BCL11A, HBS1L-MYB, and the β-globin cluster. This study aimed to determine the possible correlation between the number of SNPs and haplotypes with higher HbF levels in a cohort of patients with SCD. A positive association could explain why certain haplotypes, such as Senegal or Arab-Indian, show higher HbF levels and less severe disease.

**Methods:**

To test this hypothesis, the characterization of haplotypes was performed using the PCR-RFLP technique and genotyping of three SNPs representative of the three loci with the greatest association with HbF variation: *Xmn*I (rs7482144), BCL11A (rs4671393), and HBS1L-MYB (rs9376092).

**Results:**

We found more SNPs in haplotypes related to higher HbF than those with less HbF, although only the SNP *Xmn*I (rs7482144) showed a statistically significant association.

**Conclusion:**

We found a direct correlation between haplotypes and the number of SNPs. Haplotypes with higher levels of HbF and less severe phenotypes showed a higher number of SNPs. Thus, the Benin and Bantu haplotypes traditionally associated with poor prognosis showed the fewest mutated SNPs.

## 1 Introduction

Sickle cell disease (SCD) is characterized by complex pathophysiology largely driven by vaso-occlusion and hemolytic anemia. Patients with SCD may experience a wide range of symptoms and complications, including acute chest syndrome, infections, pulmonary hypertension, stroke, and painful vaso-occlusive crises (1).

The disease is caused by a single nucleotide transversion at codon 6 GAG > GTG (HBB: c.20A > T) (NM_000518.4) of the β-globin (*HBB*) gene, thus leading to the production of the most common hemoglobin variant worldwide, HbS, characterized by the substitution of the amino acid Glu → Val at the β6(A3) position (2). In addition, at least five different haplotypes have been characterized in the β cluster, suggesting distinct geographical origins of the same β*^S^* gene (Senegal, Benin, Bantu, Arab-Indian, and Cameroon), with haplotype differences in fetal Hb (HbF) levels (3, 4) having been documented as well. SCD (OMIM 603903) is one of the most common autosomal recessive disorders worldwide, with more than 300,000 newborns affected each year, with an expected increase to more than 400,000 by 2050 (5). In Europe, the prevalence of SCD in the 27 member states is estimated to be approximately 1 in 150, while the SCD registry in Spain shows 1,142 registered cases. Early detection, prophylactic treatments with penicillin, and vaccines have improved the quality of life and increased the life expectancy of patients with SCD, although the only curative treatment is allogeneic transplantation from a human leucocyte antigens-compatible donor (6).

Although all patients homozygous for the HbS allele have the same genotype (β*^S^*/β*^S^*), the severity of the disease may be highly variable among affected subjects, from patients with severe clinical symptoms to cases with milder symptoms. The phenotypic heterogeneity is due to both genetic and environmental determinants. The main determinants are the presence of HbF (3, 7), a modulator of the clinical and hematological features of SCD (1, 2).

Only one drug approved by the Food and Drug Administration (FDA) and European Medicines Agency (EMA) induces the production of HbF, hydroxyurea (HU); however, not all patients manage to increase HbF levels and improve. Although most patients treated with this drug respond adequately, between 10 and 20% of adults show a minimal response (8, 9).

This variability is probably due, among other causes, to baseline HbF levels varying between the haplotypes of the β cluster, heterogeneity among genes responsible for HU metabolism, and quantitative trait locus that affect the expression of γ-globin (*HBG*) genes, including *Xmn*I of the β-globin locus on chromosome 11p15, *BCL11A* on chromosome 2p15, and the intergenic region *HBS1L-MYB* on chromosome 6q23. The *Xmn*I variant (rs7482144) exerts a direct effect on the expression of the *HBG2* gene (10), while *BCL11A* (rs4671393) and *HBS1L-MYB* intergenic region (rs9376092) variants increase HbF levels by decreasing the expression of the transcriptional repressors of γ-globin chain synthesis BCL11A and MYB, respectively (7).

The main objective of this work has been to assess whether there is a direct correlation between SNPs *Xmn*I (rs7482144) located in *HBG2*, rs4671393 located in *BCL11A*, and rs9376092 located in *HBS1L-MYB* and haplotypes and whether haplotypes related to lower severity, having increased values of HbF, show a greater number of these SNPs. To meet this objective, we first analyzed the statistical association of the percentage of HbF for each SNP and then the number and frequency of SNPs in each haplotype.

## 2 Materials and methods

### 2.1 Sample collection

A total of 28 patients diagnosed with SCD (14 women and 14 men) without treatment with HU and older than 6 years (mean age 11 years) were studied. The samples were received from different Spanish regions at the San Carlos Clinical Hospital in Madrid between 2019 and 2020.

### 2.2 Hematological measurements

All patients underwent a hematometry study with reticulocyte count (Coulter LH750 Analyzer; Beckman Coulter, Brea, CA, USA) and red blood cell morphology. HbA_2_ and HbF levels were measured using high-performance liquid ion exchange chromatography (HPLC-CE; VARIANT™; Bio-Rad Laboratories, Hercules, CA, USA). Hemoglobins were studied through capillary zone electrophoresis [Sebia Capillarys Flext (Sebia, Norcross, GA)] and HPLC-CE using the short software for Bio-Rad β-thalassemia (Bio-Rad, Hercules, CA) according to manufacturer’s instructions.

### 2.3 Molecular analysis and genotyping SNPs

After genomic DNA isolation (Biorobot^®^ EZ1; Qiagen GmbH, Hilden, Germany), DNA was quantified on an Invitrogen Qubit 4 fluorometer (Thermo Scientific, Wilmington, DE, USA). The association with α-thalassemia was ruled out by screening for the most common α-thalassemia point mutations and deletions worldwide (21 overall) through multiplex PCR followed by reverse hybridization using the commercial Alpha-Globin StripAssay kit (ViennaLab Diagnostic GmbH, Vienna, Austria) with a clinical sensitivity > 90%.

The molecular characterization of HbS was performed with the β-Globin StripAssay MED (ViennaLab Diagnostic GmbH, Vienna, Austria) commercial kit, and its confirmation using automatic Sanger sequencing of the β-globin gene following the previously described protocol (11). The haplotypes of the β cluster were obtained through amplification and digestion with restriction enzymes (PCR-RFLP) according to the protocol described by Rahimi et al. (12).

The genotyping of SNPs located in the Gγ (rs7482144) and BCL11A (rs4671393) genes and the HBS1L-MYB intergenic region (rs9376092) was performed through automatic Sanger sequencing using the primers shown in Table 1.

**TABLE 1:** Primers used in SNP genotyping.

Sequence
**Gγ - XmnI (rs7482144)**
F: 5′ AAC TGT TGC TTT ATA GGA TTT T 3′
R: 5′ AGG AGC TTA TTG ATA ACC TCA GAC 3′
**BCL11A (rs4671393)**
F: 5′ ATG GGA AGA GAC CCC AAA AC 3′
R: 5′ CCT TCT GCT TCC TGT TCA CC 3′
**HBS1L-MYB (rs9376092)**
F: 5′ GAT CAC CCA TCC ATT CAT CC 3′
R: 5′ TCA CCT TCT GAT GTG AAG GAC T 3′

F = forward primer (5′→3′) and R = reverse primer (3′→5′).

### 2.4 Statistical analysis

In the descriptive study of the data, the qualitative variables are shown alongside their frequency distribution. Quantitative variables are summarized with their mean and standard deviation (SD). Quantitative variables showing an asymmetric distribution are summarized with median and interquartile range (IQR). In comparing parameters between the study groups, the association is assessed using the non-parametric Fisher test because the groups have a small sample size. A significance value of 5% is accepted for all tests. Data processing and analysis are performed using the statistical software IBM SPSS Statistics v.2°.

### 2.5 Ethical and legal aspects

All hematological indices and clinical findings were performed with the prior informed consent of the patients, and the study was approved by the Ethics Committee of the San Carlos Clinical Hospital, Madrid, Spain. All experiments were conducted in accordance with the Declaration of Helsinki.

## 3 Results

### 3.1 Hematological data

The mean value of the hematological data is shown in Table 2. The values obtained from the study of hemoglobins were: HbA2 (2.62 ± 0.52%), HbF (14.95 ± 9.13%), and HbS (81.86 ± 8.46%).

**TABLE 2:** Hematological data.

	Mean ± SD	Reference values
RBC (x 10^6^/mL)	3.11 ± 0.72	M: 4.52–5.90 F: 4.10–5.10
Hb (g/dL)	8.78 ± 1.27	M: 13.5–17.0 F: 11.6–15.0
PCV (%)	25.87 ± 4.11	M: 38.3–48.6 F: 36–45
MCV (fL)	86.36 ± 12.06	80–100.1
MCH (pg)	29.24 ± 4.72	27–32
MCHC (g/dL)	33.93 ± 1.40	33.4–35.5
RDW (%)	22.40 ± 4.06	< 15.00
Reticulocytes (%)	9.13 ± 3.60	0.5–2.5

The number of red cells (RBCs), hemoglobin (Hb), and packet cell volume (PCV) [25.87 ± 4.11%] parameters are decreased compared with normal values. Red cell distribution width (RDW) and reticulocyte count are increased. The rest of the magnitudes, mean corpuscular volume (MCV), mean corpuscular hemoglobin (MCH), and mean corpuscular hemoglobin concentration (MCHC), show values within the reference range.

### 3.2 Molecular analysis and genotyping SNPs

Haplotypes have been inferred based on the presence (+) or absence (−) of cutting at polymorphic sites by specific restriction enzymes (5′ε-*Hin*cII; 5′Gγ-*Xmn*I; GγIVSII-HicIII; AγIVSII-HicIII; 3′ψβ-*Hin*cII; and β-*Ava*II). In the study population, the African haplotypes Benin (−−−−++), Bantu (−−+−−+), Senegal (−++−++) and Cameroon (−−+++++) have been reported. The largest haplotype in the sample was Benin (70%), followed by Bantu (15%), Senegal (11%), and Cameroon (4%). The haplotypes with the highest HbF values were Benin: 16.59 ± 9.44% and Senegal: 14.52 ± 4.76%, while the Bantu haplotype showed the lowest HbF values: 5.94 ± 2.42%.

The frequency of the SNPs studied is shown in Table 3. The most frequent were the wild-type homozygous state for *Xmn*I (rs7482144) and HBS1L-MYB (rs9376092), with 88.9 and 77.8%, respectively.

**TABLE 3:** Frequency of SNP genotype in the study population.

	*Xmn*I (rs7482144)			BCL11A (rs4671393)			HBS1L-MYB (rs9376092)		
	**CC**	**CT**	**TT**	**GG**	**GA**	**AA**	**CC**	**CA**	**AA**
Frequency	24 (88.9%)	2 (7.4%)	1 (3.7%)	11 (40.7%)	11 (40.7%)	5 (18.5%)	21 (77.8%)	6 (22.2%)	0 (0%)

For *Xmn*I (rs7482144): C is the *wild-type* allele, and T is the mutated allele. For BCL11A (rs4671393): G is the *wild-type* allele, and A is the mutated allele. For HBS1L-MYB (rs9376092): C is the *wild-type* allele, and A is the mutated allele.

In all cases, the coexistence of alpha thalassemia as well as any other hemoglobinopathy was ruled out.

### 3.3 Statistical analysis

**FIGURE 1:**
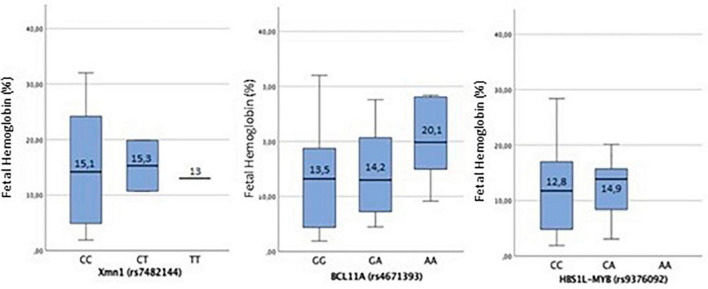
For *Xmn*I (rs7482144), there is no statistically significant association (*p* = 0.98). For the *wild-type* CC genotype, the mean ± SD of HbF is [15.00 ± 9.60%]; in CT heterozygous, it is [15.28 ± 6.47%], while in homozygous for the mutated T allele, it is [13.00 ± 0.00%]. The distribution of HbF among the genotypes of the SNP *Xmn*I (rs7482144) shows that HbF values below 10% are only found in homozygous for the *wild-type* allele (CC genotype), although HbF among these individuals has a very heterogeneous distribution. When the mutated T allele is present, both in heterozygous and homozygous, HbF levels keep above 10%. For BCL11A (rs4671393), in homozygous for the *wild-type* G allele, the mean ± SD of HbF is [13.5 ± 9.76%]; in GA heterozygous [14.24 ± 8.77%] and in homozygous for the mutated A allele [20.09 ± 8.36%]. The mean HbF values between the SNP genotypes show no statistically significant association (*p* = 0.38). The highest values of HbF among the genotypes of the SNP BCL11A (rs4671393) are reported when the mutated allele A is present, both in GA heterozygosity and in AA homozygosity, with these being much higher in the latter case. In HBS1L-MYB (rs9376092), for the homozygous for the *wild-type* C allele, the mean ± SD of HbF is [12.8 ± 8.06%], and in the CA heterozygous, it is [14.95 ± 9.13%]. The correlation of HbF between the genotypes found of the SNP HBS1L-MYB (rs9376092) shows a statistically significant association in the study population (*p* < 0.05). No homozygous individual has been found for the mutated allele. The distribution of HbF between homozygous for the *wild-type* allele (CC genotype) and heterozygous (CA genotype) is different, being higher in the latter situation.

The variation of HbF associated with the three SNPs in the three possible genotypes (homozygous for the *wild-type* allele, homozygous for the mutated allele, and heterozygous) is shown in Figure 1. Only the SNPs HBS1L-MYB (rs9376092) showed a statistically significant association in the study population (*p* = 0.002).

**FIGURE 2:**
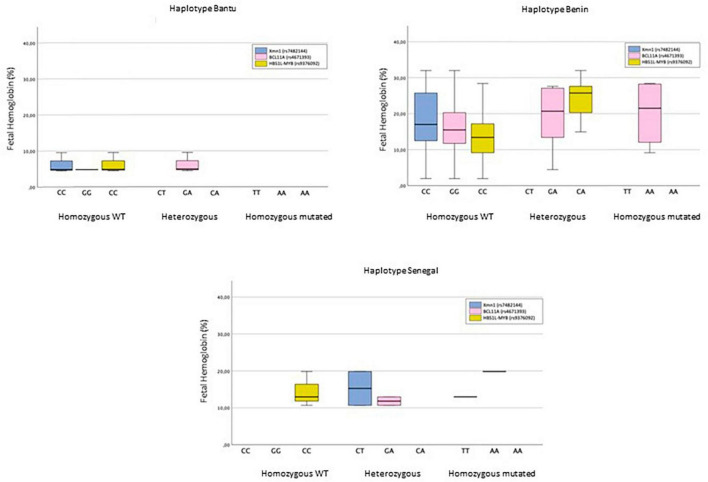
The Bantu haplotype is characterized by showing the *Xmn*I (rs7482144) and HBS1L-MYB (rs9376092) polymorphisms in homozygosity for the *wild-type* allele in 100% of cases, while the BCL11A polymorphism (rs4671393) is mostly (75%) in heterozygous individuals. HbF levels do not exceed 10%. In the Benin haplotype, the homozygous state for the *wild-type* allele is the majority in all three SNPs, accounting for 100%, 47.4%, and 68.4% of *Xmn*I (rs7482144), BCL11A (rs4671393), and HBS1L-MYB (rs9376092), respectively. Only the homozygous status for the mutated allele of SNP BCL11A (rs4671393) is reported. HbF levels are highly heterogeneous. The Senegal haplotype only shows homozygosity for the *wild-type* allele of SNP HBS1L-MYB (rs9376092). The heterozygous state is found in 66.7% of cases of both SNP *Xmn*I (rs7482144) and BCL11A (rs4671393). The homozygous genotype for the mutated allele of both SNPs constitutes 33.3%. HbF levels lie in the 10–20% range.

The number and frequency of SNPs for each haplotype are shown in Table 4. The distribution of SNPs in BCL11A and HBS1L-MYB among the different haplotypes did not produce statistically significant results. However, a statistically significant association with the SNP *Xmn*I (rs7482144) (*p* < 0.05) was observed. The distribution of HbF among SNPs in haplotypes is shown in Figure 2.

**TABLE 4:** Genotype frequency of SNPs in Bantu, Benin, and Senegal haplotypes.

	*Xmn*I (rs7482144)			BCL11A (rs4671393)			HBS1L-MYB (rs9376092)			HbF (%)	Number of chromosomes	Number of mutations
	CC (−/−)	CT (±)	TT (+/+)	GG (−/−)	GA (±)	AA (+/+)	CC (−/−)	CA (±)	AA (+/+)			
Bantu	4 (100%)	0	0	1 (25%)	3 (75%)	0	4 (100%)	0	0	5.9	8	3 (12.5%)
Benin	20 (100%)	0	0	10 (50%)	6 (30%)	4 (20%)	14 (70%)	6 (30%)	0	16.9	40	20* (16.7%)
Senegal	0	2 (66.7%)	1 (33.3%)	0	2 (66.7%)	1 (33.3%)	3 (100%)	0	0	13	6	8* (44%)
Frequency	24 (88.9%)	2 (7.4%)	1 (3.7%)	11 (40.7%)	11 (40.7%)	5 (18.4%)	21 (77.8%)	6 (22.8%)	0			
*p*-value	< 0.001			0.39			0.57					

In the Bantu haplotype, the homozygous state for the *wild-type* allele of the three SNPs is the majority, comprising 37.5% of all possible genotypes reported; 12.5% corresponds to the heterozygous state and 0% to the homozygous state for the mutated allele; 12.5% of alleles were mutated. Therefore, the low levels of HbF of this haplotype can be due to its small number of SNPs, and the fact that none of them is homozygous for the mutated allele associated with increased HbF. In the Benin haplotype, characterized by intermediate HbF values and severity, 73.3% have the *wild-type* genotype, 20% of the possible genotypes have at least one copy of the mutated allele of the SNPs, and the homozygous state for the mutated allele of the three SNPs comprises 6.6% of all reported genotypes. Overall, 16.7% of the alleles were mutated. For the Senegal haplotype, in which the homozygous state for the wild-type allele of the three SNPs comprises 33.3%, the heterozygous 44.4%, and the mutated homozygous state 22.22%, 44% of alleles for these SNPs were mutated.

*Number of mutated chromosomes.

## 4 Discussion

Sickle cell disease (SCD) has been studied for many years owing to the unexpected phenotypic heterogeneity of a disease caused by a single mutation. Several factors have been related to an improvement in complications and severity of the disease. The main and most studied ones are HbF levels, which are also subject to high heterogeneity, although there are others, such as the simultaneous inheritance of α-thalassemia or environmental factors.

For most of the time, SCD has been restricted mainly to sub-Saharan Africa, where approximately 80% of such births occur every year (5). However, it is currently found in most countries owing, among other causes, to adoptions, economic, political, or wartime migrations, thus becoming a global public health issue (3, 13).

Most treatments aim to increase HbF levels to achieve a more favorable phenotype, with HU being the main drug used. Although these treatments are only palliative and the only curative treatment is the transplantation of hematopoietic precursors, the vast majority of patients cannot access the latter because of the shortage of compatible donors, low incomes, and inadequate sanitation in countries with the highest incidence, usually coinciding with developing countries (1). Therefore, there is a growing interest in glimpsing the genetic factors responsible for the increase in HbF levels and their variability among patients and trying to genetically or pharmacologically manipulate them to achieve a less severe disease phenotype. In this regard, this study aimed to determine whether haplotypes related to higher levels of HbF and, therefore, to a disease with fewer complications show a greater number of SNPs previously described as positive modulators of HbF synthesis. The presence of these SNPs could partially explain the heterogeneity of HbF levels between haplotypes. HbF is a highly variable parameter among individuals with SCD. In the study cohort, the values ranged from 1.9 to 32%. Most of this variation (89%) is controlled by genetic factors identified over the last few years, among which the haplotypes of the β cluster and SNPs in the three quantitative trait locus studied stand out (14).

In this study, the analysis of the haplotypes of the β cluster showed four different patterns, identifying only African haplotypes, the majority being Benin, which is the most frequent in nearby countries such as Algeria or Tunisia (15). Thus, the patients studied came from or had ancestry from countries in sub-Saharan Africa. No cases have been identified for the Arab-Indian haplotype, which is more restricted to the Saudi population.

Individuals with the Bantu haplotype showed the lowest HbF levels, approximately 5%, while individuals with the highest levels were Benin and Senegal, reaching approximately 15%. HbF values in Bantu and Senegal haplotypes are consistent with levels described in other populations, while HbF values in Benin vary widely among individuals (16). The mean HbF of the Cameroon haplotype could not be calculated as it was only present in one individual (1.85%).

In our study population, probably due to sample size, the impact of β cluster haplotypes on HbF levels is not statistically significant.

Although suggesting a trend toward higher levels when the mutated allele is present, the distribution of HbF among the three genotypes of the SNP *Xmn*I (rs7482144) did not show any statistical significance since the mutated allele was identified only in two heterozygous (7.4%) subjects and one (3.7%) homozygous individual, with most individuals being homozygous for the *wild-type* allele (88.9%). Recent studies indicate that other SNPs within the β-globin cluster are more significantly associated with HbF variation than the one used in this study (17).

Regarding the genotypes of the SNP BCL11A (rs4671393) located in intron 2 of the *BCL11A* oncogene, greater variability was reported than in the previous case. The mutated allele was present in 11 heterozygous individuals (40.7%) and five (18.4%) homozygous individuals. The high frequency of the mutated allele in the sample may be because the individuals comprising it were African or of African descent, where the frequency of the mutated allele is much higher than in the rest of the populations. This SNP is associated with higher levels of HbF, and some studies have described it as the most influential, with 13% of the variability being attributed to it (17–19). In our study, genotypes containing this mutated allele show higher mean HbF levels than those homozygous for the *wild-type* allele. There is an increase in HbF in homozygous individuals for the mutated allele, where the mean HbF level is greater than 20%; however, owing to the restricted sample size, no statistically significant association could be established.

Of the SNP HBS1L-MYB (rs9376092), only two of the three possible genotypes have been reported, and no homozygous individuals for the mutated allele were reported. Despite this obstacle, it is the only variable showing a statistically significant association with HbF levels. This SNP is also associated with HbF variation in patient cohorts from other populations and healthy populations (20). It is located in block 2 of the HBS1L-MYB intergenic region, where the strongest association with HbF levels has been reported. Several studies have concluded that other SNPs (rs9399137 and rs9402686) within this block could be more related to this HbF variability (7).

There are other SNPs at cluster loci β-globin, *BCL11A*, and *HBS1L-MYB* related to variation in HbF levels. They could be used in future research to assess whether these are also found more frequently in haplotypes with higher HbF levels.

The main hypothesis of the study was that haplotypes with higher HbF levels have a higher number of mutated SNPs.

In the Bantu haplotype, no individuals with two copies of the mutated allele for any of the three SNPs were found. Therefore, the low levels of HbF of this haplotype could be due to the small number of SNPs and the fact that none of them is homozygous for the mutated allele associated with the increased HbF. In the Benin haplotype, characterized by intermediate HbF values and severity, the homozygous state for the mutated allele of the three SNPs together constitutes 6.7% of all reported genotypes, while the Senegal haplotype is the one with the highest relative frequency of SNPs in the homozygous state for the mutated allele (22.22%) from the reported genotypes.

Based on the results obtained and according to the hypothesis of the study, a direct correlation is observed between the number of SNPs homozygous for the mutated allele and haplotypes with higher levels of HbF. This distribution of SNPs could be responsible for the increase in HbF levels. Although only the SNP *Xmn*I (rs7482144) showed a statistically significant association, this result is probably due to the small sample size, so it would be necessary to expand the sample to extract more statistically robust results.

All these genetic modulators of HbF levels, and therefore of the clinic and severity of SCD, could be used as biomarkers to stratify patients based on their ability to produce HbF, with the objective of a more customized clinical and pharmacological management according to the expected phenotype. This could have implications in genetic counseling and prenatal diagnosis of patients.

The genetic SNPs described may become potential pharmacological targets to devise novel therapies that increase the level of HbF in individuals with a more severe phenotype and improve their clinical development. In this regard, strategies using *BCL11A* as a genetic target, such as silencing this gene, are being devised since this is a repressor of the synthesis of γ-globin chains. Silencing increases HbF production and corrects the disease phenotype in mouse models of SCD without affecting erythropoiesis or the expression of other genes (21). Other strategies rely on interference with *BCL11A enhancers* through genetic engineering to decrease their synthesis and thus enhance HbF production (22).

The global burden of the disease is expected to increase in the coming years, owing to improved treatment and migration to countries with higher incomes that allow for increased patient survival (5). These estimates highlight the importance of finding and exploiting genetic or pharmacological targets to improve quality of life and decrease mortality in patients with SCD.

The high heterogeneity of this disease, not yet fully explained by the genetic factors described here, implies that additional genes are involved in HbF production to be discovered. Therefore, we will better understand the genetic mechanisms underlying this disease and new candidate genes to study.

The advancements that are being performed allow us to improve the quality of life of patients, and every day we are closer to achieving a customized therapy that adjusts to the characteristics of each individual.

## 5 Conclusion

In conclusion, our study affirms that individuals exhibiting elevated HbF levels manifest a milder phenotype. Additionally, the mutated alleles of the identified SNPs are linked to an inclination for increased HbF production. The correlation between haplotypes, the quantity of SNPs, and higher HbF levels indicates that a less severe phenotype is associated with a greater number of SNPs. Notably, the Benin and Bantu haplotypes, conventionally associated with a poorer prognosis, harbor the fewest mutated SNPs. To further validate these findings, more comprehensive studies involving larger patient cohorts are warranted, especially considering the limited representation of individuals with Arab-Indian or Cameroon haplotypes in our current analysis.

## Data Availability

The datasets presented in this study can be found in online repositories. The names of the repository/repositories and accession number(s) can be found in this article/supplementary material.
